# Impact of intraoperative fluid therapy on postoperative complications following robotic-assisted minimally invasive esophagectomy (RAMIE)

**DOI:** 10.1186/s12871-025-03418-y

**Published:** 2025-11-01

**Authors:** Saeed Torabi, Dolores T. Krauss, Sandra E. Stoll, Philipp K. Omuro, Tobias Kammerer, Thomas Schmidt, Fabian Dusse, Hans A. Schlößer, Elisabeth H. Adam, Andrea U. Steinbicker, Christiane J. Bruns, Lars M. Schiffmann, Hans F. Fuchs

**Affiliations:** 1 Department of Anesthesiology and Intensive Care Medicine, Medical Faculty of Cologne University, University Hospital of Cologne, Cologne, Germany; 2 Department of General, Visceral, Thoracic and Transplant Surgery, , Medical Faculty of Cologne University, University Hospital of Cologne, Cologne, Germany

**Keywords:** Robotic-assisted minimally invasive esophagectomy (RAMIE), Intraoperative fluid therapy, Postoperative complications, Pulmonary complications, Anastomotic leakage, Postoperative atrial fibrillation (POAF), Acute kidney injury (AKI)

## Abstract

**Background:**

Robotic-assisted minimally invasive esophagectomy (RAMIE) has become an increasingly adopted approach for the treatment of esophageal cancer. However, the impact of intraoperative fluid therapy on postoperative outcomes remains poorly defined. Whereas fluid overload has been linked to pulmonary and anastomotic complications, restrictive strategies may impair tissue perfusion and organ function. This study investigates the association between intraoperative fluid balance and postoperative morbidity in patients undergoing RAMIE.

**Methods:**

We conducted a retrospective single-center cohort study including 254 consecutive patients who underwent elective RAMIE between 2019 and 2024. Intraoperative fluid balance was calculated in mL/kg/h and analyzed as a continuous variable. Primary endpoints included pulmonary complications, anastomotic leakage, postoperative atrial fibrillation (POAF), and acute kidney injury (AKI). Secondary endpoints comprised ICU length of stay (LOS), postoperative delirium, delayed gastric emptying (DGE), and complication severity according to the Clavien-Dindo classification. Multivariable regression models were adjusted for age, sex, BMI, and ASA status.

**Results:**

Pulmonary complications (23.2%) were significantly associated with higher intraoperative fluid volumes (mean: 5.2 vs. 4.4 ml/kg/h; *p* = 0.027; OR: 1.24, 95% CI: 1.05–1.46). Anastomotic leakage (18.5%) exhibited an inverted U-shaped relationship, with the highest risk at fluid levels of 4.7–8.1 ml/kg/h). POAF (16.1%) and AKI (5.5%) were not significantly associated with fluid volume in multivariable analysis. POAF showed no significant association with intraoperative fluid volume in adjusted models. Predicted probabilities illustrated a fivefold increase in pulmonary risk across the 0 to 10 ml/kg/h range, whereas POAF declined steadily over this interval. Postoperative delirium showed a trend toward association with fluid volume (OR: 1.34; *p* = 0.056), while DGE, ICU-LOS, and major complications demonstrated no significant associations. Subgroup analyses suggested stronger associations between fluid volume and pulmonary complications in elderly patients, and a more pronounced POAF risk in males, indicating potential effect modification by age and sex.

**Conclusion:**

Intraoperative fluid volume during RAMIE is variably associatiated with postoperative outcomes. While higher volumes are linked to increased pulmonary morbidity, lower volumes may predispose patients to arrhythmias. Anastomotic complications appear to peak at moderate fluid levels. These findings challenge binary fluid strategies and support a more individualized, risk-adapted approach to intraoperative fluid management in esophageal surgery.

## Introduction

Esophagectomy remains a high-risk surgical procedure with substantial morbidity and mortality, necessitating meticulous perioperative management to optimize patient outcomes. Among important non-surgical factors with influence on postoperative recovery, intraoperative fluid therapy plays a pivotal role to maintain hemodynamic stability, preserve organ perfusion, and mitigate complications such as acute kidney injury (AKI), pulmonary complications, and anastomotic leakage [1].

Recent studies have explored the association between intraoperative fluid therapy and postoperative morbidity in patients undergoing esophagectomy [2]. A randomized controlled trial evaluated staged goal-directed fluid therapy (GDT) and demonstrated that GDT can effectively reduce postoperative pulmonary complications while intraoperative hemodynamic parameters were improved [3]. In contrast, an observational study focusses on volume management in robotic-assisted minimally invasive esophagectomy (RAMIE) and found no substantial differences in postoperative outcomes between -restrictive and liberal fluid strategies [4].

The impact of intraoperative fluid therapy on patient outcomes remains complex, as both liberal and restrictive regimens carry potential risks [4–6]. Excessive fluid administration has been associated with a higher incidence of respiratory complications, including acute respiratory distress syndrome (ARDS) and pneumonia [7]. One study reported that elevated intraoperative fluid volumes have been associated with increased rates of ARDS and in-hospital mortality [8]. Conversely, although fluid restriction may mitigate the risk of pulmonary complications, it raises concerns regarding renal dysfunction and the potential need for vasopressor support to maintain hemodynamic stability [4].

Given these conflicting risks, intraoperative fluid management has moved beyond a purely physiological intervention and is now embedded in broader perioperative strategies such as ERAS, Within these protocols, goal-directed fluid management is an integral component of enhanced recovery after surgery (ERAS) protocols, which aim to optimize perioperative care [9]. The adoption of goal-directed fluid management – with integrated dynamic hemodynamic monitoring and patient-specific parameters - offers a promising strategy to achieve an optimal balance between adequate fluid administration and complication prevention. Despite the recognized importance of fluid management [10], there is an ongoing debate regarding the optimal fluid strategy. To date, it remains unclear whether restrictive, liberal, or goal-directed approaches offer the greatest benefit to minimize postoperative complications and to improve surgical outcomes. This retrospective study therefore aims to evaluate the impact of intraoperative fluid therapy on postoperative outcomes in patients undergoing esophagectomy. Thereby a contribution to the evolving body of evidence about perioperative fluid optimization strategies is pursued.

## Methods and statistics

### Study design and setting

This retrospective single-center cohort study was conducted at Cologne University, the University Hospital of Cologne, Germany, in collaboration between the Departments of Anesthesiology and Intensive Care Medicine and General, Visceral, Thoracic, and Transplant Surgery. The study included adult patients from a surgical database, who underwent RAMIE between March 2019 and June 2024 and received postoperative care at one of the hospital’s Intensive Care Units.

#### Study hypothesis

We hypothesized that intraoperative fluid balance, expressed in ml/kg/h, is associated with greater occurrence of major postoperative complications in patients undergoing RAMIE. Furthermore, we assumed that the relationship between fluid volume and postoperative outcomes is potentially non-linear and complication-specific, so that a differentiated analytical approach rather than simplistic liberal versus restrictive categorization is required.

#### Primary endpoint

The primary objective of this study was to investigate the association between intraoperative fluid balance and the incidence of postoperative complications:

- Postoperative pulmonary complications, including pneumonia, respiratory insufficiency, and pleural effusion that require intervention.
- Anastomotic complications, including anastomotic leakage or conduit ischemia.
- Postoperative atrial fibrillation (POAF) within seven postoperative days.
- Acute kidney injury (AKI), defined according to KDIGO criteria.

#### Secondary endpoints

- ICU length of stay (ICU-LOS), analyzed using log-transformed duration.
- Postoperative delirium, defined as clinically diagnosed and documented episodes that required pharmacological management.
- Delayed gastric emptying (DGE), diagnosed based on clinical and/or radiological findings.
- Severity of postoperative complications, classified according to the Clavien-Dindo classification system, with a focus on major complications (Grade ≥ IIIa).

Each outcome was analyzed using multivariable regression models adjusted for relevant clinical confounders (age, sex, BMI, ASA score), with special consideration for potential non-linear effects by including second-order polynomial terms where appropriate.

#### Inclusion criteria

This study included all adult patients who underwent elective, minimally invasive robotic-assisted Ivor-Lewis esophagectomy, characterized by either robotic or laparoscopic gastrolysis combined with robotic transthoracic esophagectomy.

#### Exclusion criteria

Patients were excluded from the study based on the following criteria:

- Emergency esophagectomy for trauma or Boerhaave syndrome.
- McKeown esophagectomy with cervical anastomosis.
- Benign indications such as achalasia.
- Open esophagectomy.
- Intraoperative conversion from robotic-assisted to open esophagectomy.
- Missing anesthesia protocols or incomplete intraoperative data.
- Intraoperative administration of gelatine-based colloid solutions.

#### Surgical technique

A detailed treatment pathway of patients with esophageal cancer at our centre has been published previously [11]. As most patients present with locally advanced tumors, a multimodal treatment follows, and surgery is performed 4–6 weeks after completion of neoadjuvant treatment and restaging diagnostics. The current standard procedure is a totally minimally invasive Ivor Lewis esophagectomy with reconstruction using a gastric conduit and high intrathoracic anastomosis, performed with the DaVinci Xi robotic surgical system (Intuitive Surgical Inc, Sunnyvale, CA. USA). The abdominal part may be performed either robotic- laparoscopic or manual-laparoscopic. Our standardized surgical technique has beend described in detail elsewhere [11].

The patient selection ensured a homogeneous patient cohort undergoing a standardized surgical approach (RAMIE) and postoperative critical care.

### Data collection

All adult patients who underwent RAMIE for esophageal cancer between 2019 and 2024 at the University Hospital of cologne were screened for study inclusion using Datawarehouse eisTIK (KMS GmbH, Unterhaching, Germany). Perioperative data were retrospectively collected and documented in Microsoft Excel (Microsoft Corporation, Redmond, USA). Electronic data sources included the hospital´s electronic medical records and paper-based documentation from patients` charts, and anesthesia records. Twenty-two patients had incomplete components to derive ml/kg/h balance and were excluded from analyses that specifically used balance; they were retained for analyses using administered volume.

Data extraction included:

- Demographic Data: age, sex, and relevant comorbidities.
- Surgical variables: surgical approach (robotic assisted Ivor-Lewis esophagectomy), duration of surgery, duration of one-lung ventilation, intraoperative blood loss, volume of intraoperative fluid administration and total intraoperative fluid balance including urinary output and transfusions.
- Postoperative outcomes: incidence of postoperative atrial fibrillation, pulmonary complications anastomotic leakage, ICU length of stay, acute kidney injury, and in-hospital mortality.

The following endpoints and clinical variables were defined as follows:

#### The intraoperative fluid balance

Intraoperative fluid balance was expressed in ml/kg/h and calculated as the total amount of intraoperatively administered balanced crystalloid solutions minus urinary output and blood loss, divided by body weight and operative duration in hours.

#### Acute kidney injury (AKI)

Postoperative AKI was defined according to the Kidney Disease: Improving Global Outcomes (KDIGO) criteria [12], based on changes in serum creatinine and urine output. All patients in this study received balanced crystalloid solutions intraoperatively.

#### Postoperative atrial fibrillation (POAF)

POAF was defined as a hemodynamically significant postoperative atrial fibrillation (AF) occurring within 7 days after surgery with the day of surgery considered day zero. The diagnosis was confirmed by electrocardiogram (ECG) and required either medical or electrical intervention.

#### Pulmonary complications

Postoperative pulmonary complications were defined as the occurrence of pneumonia, clinically significant atelectasis requiring non-invasive ventilation (NIV), or pleural effusions requiring chest tube placement. These complications were further characterized by the presence of partial or global respiratory insufficiency.

### Statistical analysis

Anonymized data were transferred into Microsoft Excel. Statistical analyses were performed using R version 4.4.3 (R Core Team. *R: A Language and Environment for Statistical Computing*. R Foundation for Statistical Computing, Vienna, Austria).

analyses were conducted using χ² tests for categorical variables and Kruskal–Wallis tests for non-normally distributed continuous variables. A *p-value < 0.05 was considered statistically significant. Data are displayed as mean [25.−75. percentile] or relative frequency % (n = absolute frequency). Continuous variables were presented as mean, standard deviation, median, minimum and maximum. Categorical variables were summarized using absolute and relative frequenciesy. Differences in mean intraoperative fluid volumes between complication groups were assessed using Welch’s t-test. To visualize the relationship between intraoperative fluid volume (treated as continuous variable) and binary outcomes (complication: yes/no), fluid volume was categorized into five equally spacedintervals. The relative frequency of complications was then plitted across these catregories.

For binary outcomes logistic regression models were used. Specification of the regression models were tested for non-linearities in the relationship between fluid volume and outcomes by including a second-order polynomial term in the regression equations (y = x + x²). If the second-order polynomial specification did not result in a better model fit (Likelihood-Ratio test), a linear specification (y = x) was applied. All regression models were adjusted for potential confounders, including age, sex, BMI, and ASA classification. Predicted probabilities from regression models were calculated based on standardized covariate values: age = 62.75, sex = male, BMI = 26.07, ASA = II.

This study reexamines the complex relationship between intraoperative fluid volume and postoperative complications using a methodological approach that deliberately avoids dichotomizing or categorizing the continuous variable of fluid volume [13].

## Results

A total of 254 patients were included in the study. The median age was 63 years (Interquartile range [IQR] 34–83), and the median body mass index (BMI) was 25.4 kg/m² (IQR 15.6–43.4). The majority of patients were male (82.7%).

Common comorbidities included hypertension in 42.5%, peripheral arterial disease (PAD) in 48%, and diabetes mellitus in 12.6% of patients. Preexisting atrial fibrillation and coronary artery disease were present in 7.9% and 8.7% of patients, respectively. Chronic obstructive pulmonary disease (COPD) was also documented in 8.7% of the cohort.

Most patients (89.8%) received neoadjuvant chemotherapy prior to surgery. A reduced preoperative glomerular filtration rate (GFR below 90 ml/min/m^2^), indicating renal impairment, was observed in 5.1% of patients. According to the American Society of Anesthesiologists (ASA) classification, 21.7% of patients were classified as ASA I, 58.7% as ASA II, and 19.3% as ASA III. (Table 1).

**Table 1:** Preoperative characteristics of all patients (n = 254). BMI = body mass index. COPD: chronic obstructive pulmonary Disease. ASA = American society of Anesthesiology. PAD: peripheral arterial Disease. *Values are presented as median (interquartile range) or n = absolute frequency (relative frequency %)

Patients` Characteristics	Median (IQR) or *n* (%)
Age (years)	63.0(34–83) *
BMI (kg/m^2^)	25.4(15.6–43.4) *
Gender (Male/female)	210 (82.7%)/44(17.3%)
Hypertension, n (%)	108 (42.5%)
PAD, n (%)	122 (48%)
Preexisting atrial Fibrillation, n (%)	20 (7.9%)
Perioperative Kidney Impairment	13 (5.1%)
Coronary artery disease, n (%)	22 (8.7%)
Diabetes mellitus, n (%)	32 (12.6%)
COPD, n (%)	22 (8.7%)
Neoadjuvant Chemotherapy, n (%)	228 (89.8%)
ASA-Classification, n (%)	
I	55 (21.7%)
II	149 (58.7%)
III	49 (19.3%)

The median operative duration was 342 min (IQR 307–396), with a median one-lung ventilation time of 175 min (IQR: 75–380). Patients received a median volume of 2500 ml crystalloid solution (IQR: 300–6500) intraoperatively, and the median intraoperative blood loss was 200 ml (IQR: 50–800). Median urinary output during surgery was 450 ml (IQR: 10–1900).

The resulting fluid balance was 2080 ml (IQR: −1100 to 4910), corresponding to a median weight-adjusted intraoperative fluid balance of 4.28 ml/kg/h (−2.12 to 14.9). (Table 2).

**Table 2:** Intraoperative Parameter. Values are presented as median (interquartile range, IQR)

Intraoperative Parameter	Median (IQR)
Operative Duration (min)	342 (307–396)
One-Lung Ventilation (min)	175 (75–380)
Crystalloid Solution (ml)	2500 (300–6500)
Blood loss (ml)	200 (50–800)
Urine output (ml)	450 (10–1900)
Fluid balance (ml)	2080 (−1100-4910)
Fluid balance (ml/kg/h)	4.28 (−2.12-14.9)
Noradrenalin-Dosis upon ICU admission (µg/kg/min)	0.05 (0.01–0.80)

The median length of ICU stay was 2 days [2–4], and the median in-hospital stay was 14 days[12–21].

A range of postoperative complications was observed in the study population. Anastomotic leakage or conduit ischemia occurred in 47 patients (18.5%), while POAF was identified in 41 cases (16.1%). Pulmonary complications were frequent adverse events, affecting 59 patienÄ’ts (23.2%) and included pneumonia, pleural effusions and respiratory insufficiency. Delayed gastric emptying was the most common complication, reported in 63 patients (24.8%). Acute kidney injury was diagnosed in 14 patients (5.5%), and postoperative delirium occurred in 15 patients (5.9%). (Table 3)

**Table 3:** Incidence of postoperative complications

Postoperative complications	*n* (%)
Anastomotic leakage or Conduit ischemia	47 (18.5%)
Postoperative atrial fibrillation	41 (16.1%)
Acute kidney Injury	14 (5.5%)
Pulmonary complications	59 (23.2%)
Delayed gastric emptying	63 (24.8%)
Postoperative Delirium	15 (5.9%)

**Fig. 1:**
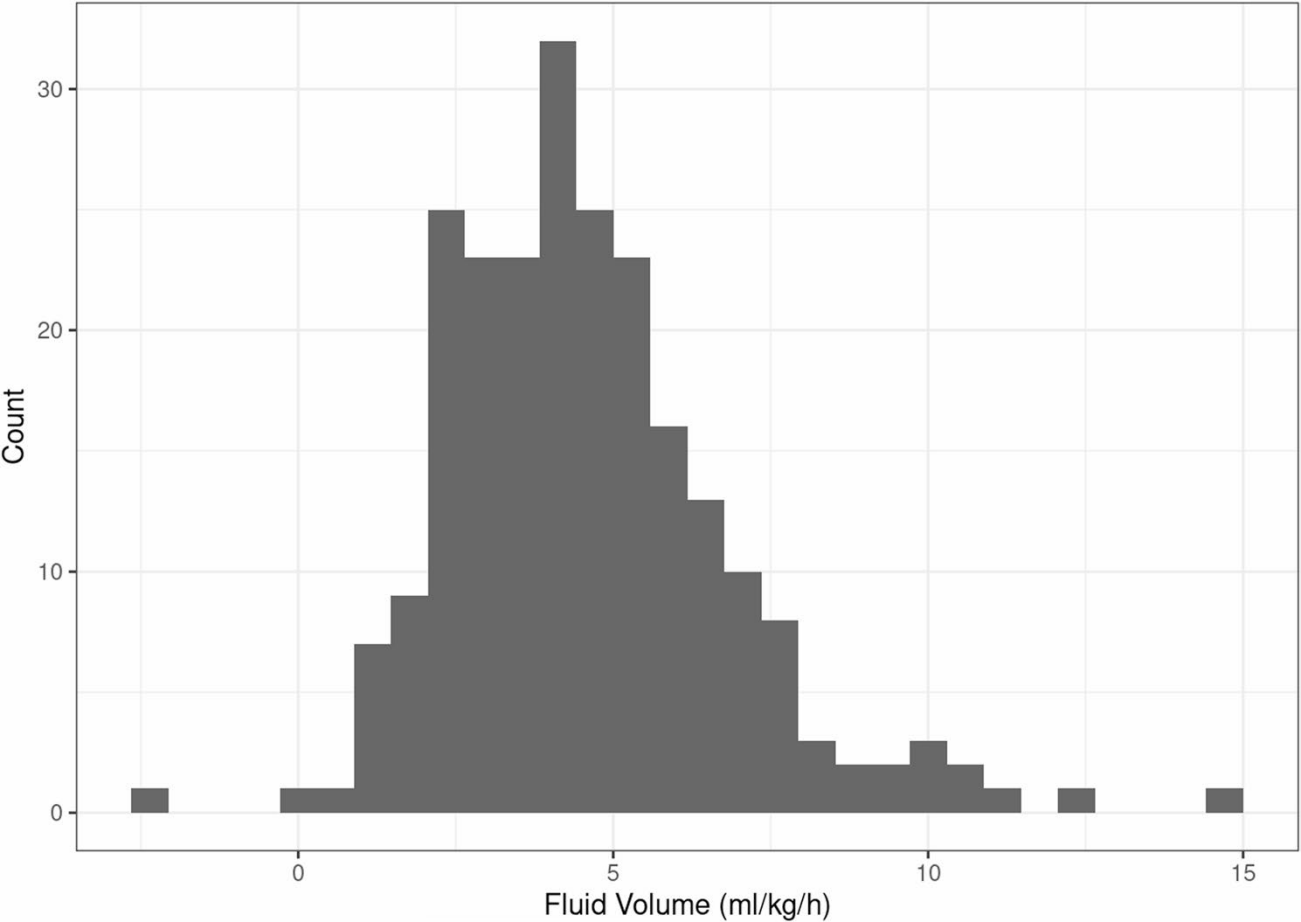
Distribution of intraoperative fluid volume per bodyweight and time (ml/kg/h). The x-axis represents the distribution of intraoperative fluid balance, while the y-axis indicates the corresponding number of patients

Most patients received intraoperative fluid therapy between approximately 3 and 7 ml/kg/h, with a median of 4.28 ml/kg/h (range: −2.12 to 14.9). The distribution appeared approximately normal with a slight right skew, indicating a small number of patients received substantially higher fluid volumes. Outliers beyond 10 ml/kg/h were rare. (Fig. 1)

**Fig. 2:**
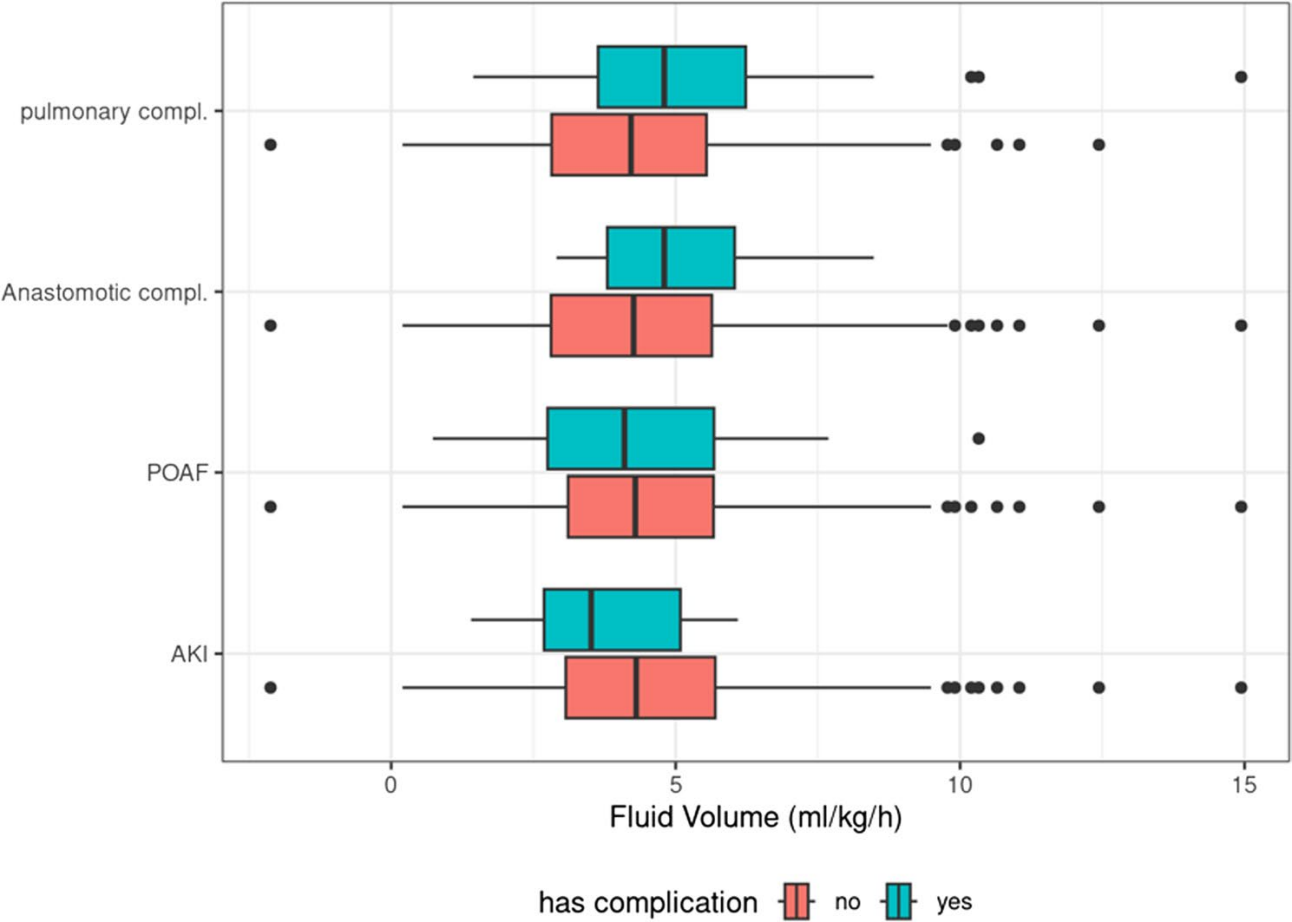
Boxplots of intraoperative fluid administration stratified by the presence (green) or absence (red) of specific postoperative complications. The y-axis lists the complications: AKI (acute kidney injury), POAF (postoperative atrial fibrillation), pulmonary complications, and anastomotic leakage. The x-axis represents the intraoperative fluid administered in ml per kilogram body weight per hour (ml(kg/h)

Figure2 shows boxplots of intraoperative fluid administration stratified by the presence (green) or absence (red) of the following postoperative complications:

- AKI (acute kidney injury).
- POAF (postoperative atrial fibrillation).
- Anastomotic complications.
- Pulmonary complications.

When comparing intraoperative fluid administration between patients with and without postoperative complications, a statistically significant difference was observed for pulmonary complications. Patients who developed pulmonary complications received a higher mean intraoperative fluid volume (5.2 ml/kg/h) compared to those without such complications (4.4 ml/kg/h;, *p =* 0.027, Table 4).

**Table 4:** Results of welch’s t-tests comparing intraoperative fluid volume (ml/kg/h) between patients with presence and absence of specific postoperative complications. AKI (acute kidney injury), POAF (postoperative atrial fibrillation)

Postoperative Complication	Mean volume (SD) Absence of complication	Mean volume (SD) Presence of complication	*p*-value
AKI	4.7 (2.3)	3.7 (1.6)	n.s.
POAF	5.2 (2.4)	4.4 (2.0)	n.s.
Anastomotic complications	4.5 (2.3)	5.1 (1.6)	n.s.
Pulmonary complications	4.4 (2.1)	5.2 (2.4)	0.027

In patients who developed anastomotic leakage, the mean intraoperative fluid administration trended to be numerically higher (5.1 ml/kg/h) compared to those without leakage (4.5 ml/kg/h, n.s.). There was also no difference in intraoperative fluid administration between patients who developed POAF (4.4 ml/kg/h) and those who did not (5.2 ml/kg/h) and in patients who developed postoperative AKI (3.7 ml/kg/h) compared to those without AKI (4.7 ml/kg/h) (Table 4).

**Fig. 3:**
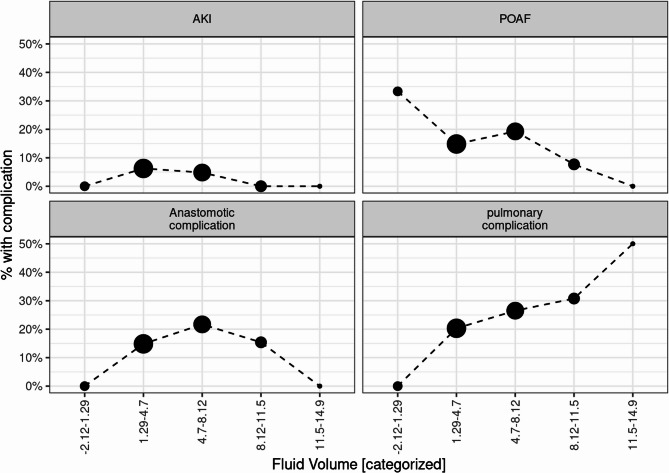
Dot-line plots illustrate the association between intraoperative fluid balance (categorized into five equally spaced quintiles in ml/kg/h) and postoperative complications. The size of each dot reflects the number of patients within each fluid volume category. The four panels display the percentage of patients experiencing one of the following complications: acute kidney injury (AKI), postoperative atrial fibrillation (POAF), pulmonary complications, and anastomotic complications

To explore the relationship between intraoperative fluid volume and the occurence of postoperative complications, intraoperative fluid balance was stratified into five equally spaced intervals (Table 5). The percentage of patients experiencing each complication was then analyzed and visualized in Figure 3.

**Table 5:** Categorization of intraoperative fluid administration (ml/kg/h) into five equally spaced- intervals (quintiles) and corresponding patient distribution

Intraoperative Fluid balance Quintiles ml/kg/h	*N*
−2.12-1.29	6
1.29–4.7	128
4.7–8.12	83
8.12–11.5	13
11.5–14.9	2
Missing Data	22

The incidence of acute kidney injury (AKI) ranged from approximately 0% to 10% across all fluid volume categories and showed minimal variation between groups.

The indidence of POAF was highest in the lowest fluid volume category (−2.12 to 1.29 ml/kg/h), with approximately 30% of patients affected. In the higher fluid volume groups, the incidence progressively declined, reaching below 10% in the highest category (11.5–14.9 ml/kg/h).

The incidence of anastomotic complications increased across the lower to the mid-range fluid volume categories, peaking at approximately 25% in the 4.7–8.12 ml/kg/h group. In higher volume categories the incidence declined. This suggests a possible inverted U-shaped relationship between fluid administration and anastomotic outcomes.

Notably, the incidence of pulmonary complications increased with higher fluid volume categories, rising from near 0% in the lowest group to almost 40% in the highest category.

These findings highlight a differential relationship between fluid volume and postoperative complications. While higher volumes may be protective against POAF, they appear to increase the risk of pulmonary complications. Anastomotic outcomes may be most vulnerable at intermediate fluid levels, and AKI incidence remained unaffected across the observed volume spectrum.

To assess the independent association between intraoperative fluid administration and postoperative complications, we performed multivariable logistic regression analyses for each binary outcome (Table 6). Fluid volume was modeled either as a linear term or, when appropriate, as a second-order polynomial term (volume²), based on model fit and the shape of the relationship observed in exploratory analyses. All models were adjusted for clinically relevant covariates, including age, gender, BMI, and ASA classification, to account for potential confounding. Covariates were selected based on their plausible clinical association with both intraoperative fluid administration and postoperative outcomes.

**Table 6:** Multivariate analysis for primary endpoints. BMI = body mass index. ASA = American society of Anesthesiology. Acute kidney injury (AKI), postoperative atrial fibrillation (POAF). CI = Confidence interval. Values in bold indicate statistical significance (p < 0.05).

	Pulmonary complication			Anastomotic complication			POAF			AKI		
Predictors	Odds Ratios	CI	P	Odds Ratios	CI	p	Odds Ratios	CI	p	Odds Ratios	CI	p
(Intercept)	0.01	0.00–0.29	**0.012**	0.00	0.00–0.01	**< 0.001**	0.01	0.00–1.27	0.067	0.00	0.00–0.12	**0.016**
Fluid Volume	1.24	1.05–1.46	**0.012**	4.88	1.85–17.20	**0.005**	0.90	0.74–1.09	0.290	0.94	0.64–1.37	0.748
Fluid Volume²				0.88	0.79–0.96	**0.016**						
Age	1.02	0.98–1.06	0.371	1.04	0.99–1.09	0.113	1.04	1.00–1.09	0.087	1.04	0.96–1.13	0.335
Gender [female]	1.09	0.47–2.39	0.832	0.77	0.26–1.95	0.600	1.33	0.50–3.29	0.544	0.544	0.13–4.71	0.987
BMI	1.07	0.98–1.16	0.123	1.09	0.98–1.20	0.096	0.98	0.89–1.07	0.662	0.662	1.02–1.35	**0.027**
ASA [II]	1.21	0.55–2.85	0.645	0.56	0.24–1.37	0.194	3.49	1.15–15.22	**0.050**	**0.50**	0.27–8.54	0.817
ASA [III]	1.24	0.46–3.38	0.668	0.77	0.27–2.19	0.630	3.17	0.85–15.32	0.107	0.107	0.16–9.36	0.913

Pulmonary complications were positively and significantly associated with intraoperative fluid administration (OR: 1.24, 95% CI: 1.05–1.46, *p* = 0.012). Specifically, each additional ml/kg/h of fluid administered was associated with a 24% increase in the odds of pulmonary events, suggesting a dose-dependent relationship.

For anastomotic leakage, a non-linear (second-order polynomial) model best captured the relationship. The predicted risk was highest at moderate fluid volumes (~ 5–8 ml/kg/h), consistent with an inverted U-shaped association. The likelihood of anastomotic leakage was lower at both the lower and higher ends of the fluid distribution.

In contrast, postoperative atrial fibrillation (POAF) and acute kidney injury (AKI) were not associated with intraoperative fluid volume in the multivariable models, although the direction of the estimated effect was negative in both cases.

Regarding covariates, ASA classification emerged as a strong independent predictor for POAF: patients classified as ASA II had a borderline signifikant higher risk compared to those classified as ASA I (OR: 3.49, 95% CI: 1.15–15.22, *p* = 0.050). Additionally, higher BMI was significantly associated with an increased risk of AKI (*p* = 0.027), highlighting the relevance of patient-specific factors in modulating susceptibility to postoperative outcomes.

**Fig. 4:**
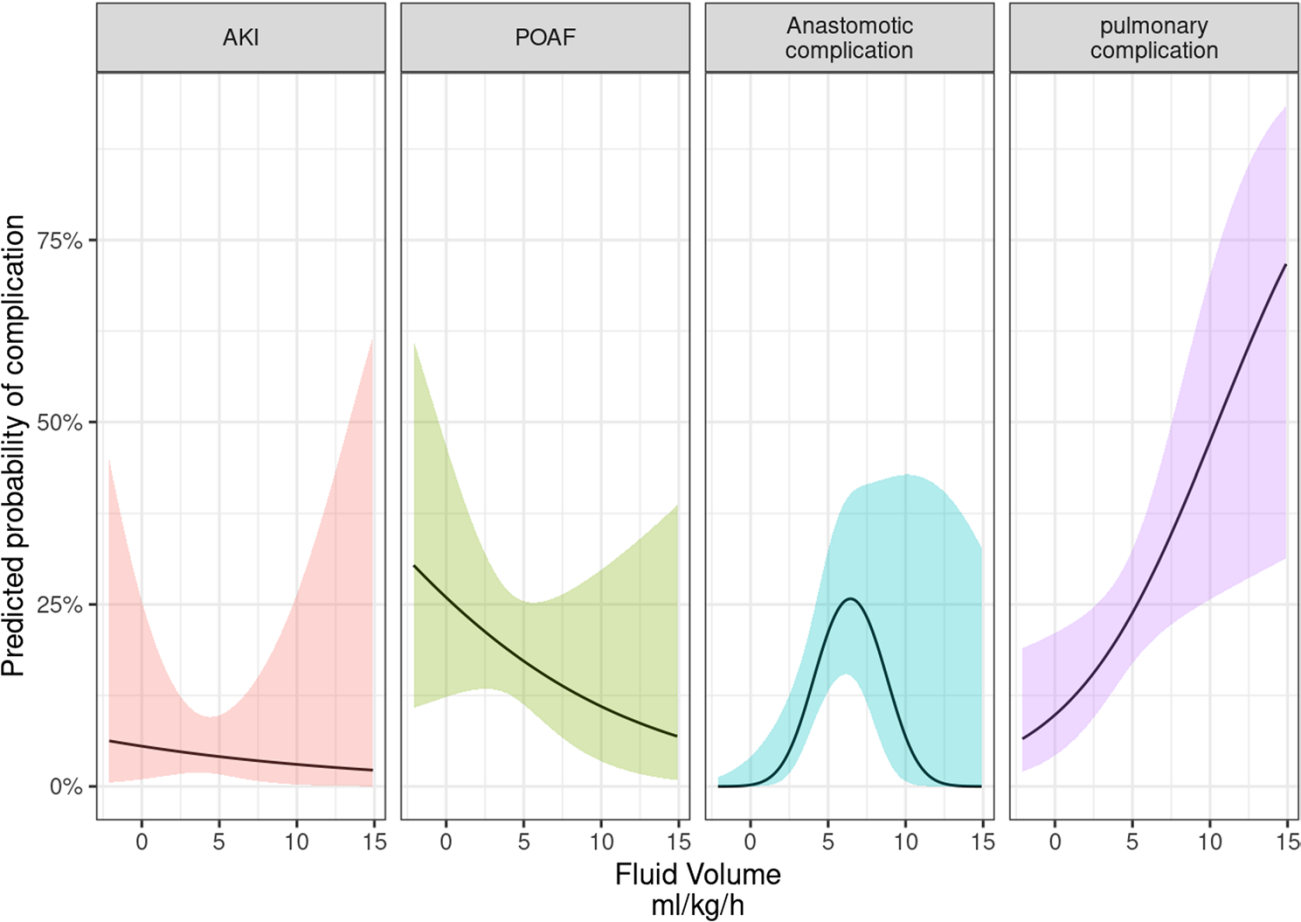
Predicted probability of postoperative complications across intraoperative fluid administration levels (adjusted for age = 62.75, sex = male, BMI = 26.07, and ASA classification = II). Black linse represent point estimates; shaded areas indicate 95% confidence intervals. Panels depict predicted risks for acute kidney injury (AKI, postoperative atrial fibrillation (POAF) anastomotic complications, and pulmonary complications

To further illustrate the association between intraoperative fluid administration and postoperative complications, we plotted predicted probabilities derived from the multivariable logistic regression models (Fig. 4). The analysis was adjusted for relevant confounders, including age, sex, BMI, and ASA classification.

The predicted probability of AKI remained low across the observed range of intraoperative fluid volumes, with 5.5% (95% CI: 1%; 25.4%) at 0 ml/kg/h and 3.0% (95% CI: 0.3%, 26.4%). No consistent association with fluid volume was observed, as indicated by the shallow negative slope and wide confidence intervals.

The predicted probability of POAF decreased from 26% (95% CI: 12.3%; 46.7%) at 0 ml/kg/h to 10% (95% CI: 3.5%; 29.8%) at 10 ml/kg/h; however, this association did not reach statistical significance.

For anastomotic complications, the relationship follows an inverted U-shape. The predicted probability was very low at minimal fluid levels – 0.02% (95% CI: <0.01%−4.1%) at 0 ml/kg/h – but increased at intermediate levels, peaking at 25.8% (95% CI: 15.3%−40%) at 6.4 ml/kg/h. With further increases in fluid volume, the probability declined again, reaching 6.8% (95% CI: 0.7%−42.8%) at10 ml/kg/h.

Pulmonary complications were more likely at higher levels of intraoperative fluid administration, with a predicted probability of 9.8% (95% CI: 4.3%−21.1%) at 0 ml/kg/h and 47.7% (95% CI: 25.7%−70.1%) at 10 ml/kg/h. This statistically significant increase corresponds to nearly a fivefold rise in absolute risk over the observed fluid range.

### Secondary endpoints

We conducted multivariable logistic and linear regression models to investigate the association between intraoperative fluid administration and selected secondary outcomes, including postoperative delirium, delayed gastric emptying (DGE), ICU length of stay (ICU-LOS), and complication severity as classified by the Clavien-Dindo classification (Table 7). All models were adjusted for age, sex, BMI, and ASA classification to account for potential confounding.

**Table 7:** Multivariate analysis for secondary endpoints. BMI = body mass index. ASA = American society of Anesthesiology. ICU length of stay (ICU-LOS) delayed gastric emptying (DGE). Clavien-Dindo classification was dichotomized as major (grade > IIIa) versus minor (≤ II). Values in bold indicate statistical significance (p < 0.05).

	ICU LOS			Delirium			DGE			Clavien Dindo “major”		
Predictors	Estimates	CI	p	Odds Ratios	CI	p	Odds Ratios	CI	p	Odds Ratios	CI	p
(Intercept)	0.20	1.00–1.40	n.s.	0.00	0.00–0.43	**0.038**	3.00	0.07–131.55	n.s.	0.11	0.00–8.32	n.s
Volume rel	0.03	0.02–0.08	n.s.	1.34	0.99–1.83	n.s.(0.06)	0.90	0.76–1.05	n.s.	0.95	0.78–1.14	n.s.
Age	0.01	0.01–0.02	n.s.	1.04	0.97–1.12	n.s.	0.98	0.94–1.02	n.s.	1.01	0.96–1.05	n.s.
Gender[female]	0.23	0.02–0.49	n.s. (0.07)	0.61	0.08–2.72	n.s.	1.96	0.91–4.12	n.s. (0.08)	3.00	1.32–6.73	**0.008**
BMI	0.02	0.01–0.04	n.s.	1.08	0.90–1.27	n.s.	0.97	0.89–1.04	n.s.	1.03	0.95–1.13	n.s.
ASA[II]	−0.11	0.36–0.13	n.s.	1.12	0.24–7.93	n.s.	1.36	0.62–3.18	n.s.	0.51	0.23–1.15	n.s
ASA [III]	−0.06	0.36–0.24	n.s.	2.03	0.35–15.79	n.s.	1.82	0.70–4.90	n.s.	0.42	0.14–1.21	n.s.

A trend towards an increased risk of postoperative delirium was observed with higher intraoperative fluid volumes. Fluid administration (ml/kg/h) was positively associated with delirium (OR: 1.34, 95% CI: 0.99–1.83), close to statistical significance (*p* = 0.056). Age, sex, and other covariates were not independently associated with delirium in this model. Intraoperative fluid volume and the occurrence of DGE (OR: 0.90, 95% CI: 0.76–1.05, *p* = 0.177) were not associated. Female sex showed a non-significant trend towards increased odds of DGE (OR: 1.96, *p* = 0.079).

In the linear regression model with log-transformed ICU-LOS, intraoperative fluid administration was not significantly associated with lengths of ICU stay duration, age, sex, BMI, or ASA classification.

The likelihood to experience major postoperative complications (Clavien-Dindo grade ≥ IIIa) was independent of intraoperative fluid(OR: 0.95; 95% CI: 0.78–1.14; p = n.s.). Female sex emerged as a significant predictor of higher-grade complications (OR: 3.00; 95% CI: 1.32–6.73; *p = 0.008). Female patients exhibited a significantly higher incidence of severe postoperative complications (Clavien-Dindo ≥ III) compared to males (31% vs. 18%, *p* = 0.008). In multivariable logistic regression, the association between intraoperative fluid volume and severe complications was more pronounced in females (adjusted OR per ml/kg/h: 1.41 [95% CI 1.12–1.84]) than in males (OR: 1.09 [0.88–1.35], *p* = 0.035).

## Discussion

This study investigated the association between intraoperative fluid therapy and postoperative complications in 254 patients undergoing robotic assisted minimally invasive esophagectomy (RAMIE). Our findings revealed complex associations between intraoperative fluid administration and specific postoperative outcomes, underscoring the importance of individualized perioperative fluid management in RAMIE procedures.

Nonetheless, this study has several limitations. Its retrospective design and small sample size limits the ability to establish causal conclusions, and the single-center setting may constrain the generalizability of the results. Moreover, residual confounding cannot be excluded with respect to intraoperative hemodynamic parameters or postoperative fluid management, which were not fully captured. Given the relatively low incidence of certain complications, such as acute kidney injury (AKI) and anastomotic leakage, larger multicenter studies are warranted to confirm and strengthen these findings.

Our analysis demonstrated an association between higher intraoperative fluid balance and an increased incidence of postoperative pulmonary complications. This finding is consistent with previous studies on minimally invasive esophagectomy. In a retrospective analysis of 1,040 patients, D’Souza et al. also reported a significant association between increased intraoperative fluid administration and postoperative pulmonary complications, including pneumonia and acute respiratory distress syndrome (ARDS) [7]. Notably, the mean intraoperative fluid volume in their cohort was substantially higher than in ours (11.92 mL/kg/h versus 4.28 mL/kg/hThe potential role of fluid exposure therefore is as a modifiable risk factor for pulmonary morbidity following esophagectomy. Similarly, Hikasa et al. reported comparable findings to our study: they demonstrated that greater intraoperative fluid overload was independently associated with a higher incidence of postoperative complications [14]. Van Dessel et al. highlighted that patients with fluid overload had a 10.24-fold increased risk to develop postoperative respiratory complications compared to those without fluid overload or vasopressor dependency [8]. In two additional studies, not only intraoperative fluid administration, but also high cumulative postoperative fluid balance was found to be associated with an increased risk of pulmonary complications [2, 15].

Surgical technique also appears to significantly impact pulmonary outcomes. The ROBOT trial demonstrated clear advantages for minimally invasive techniques over open esophagectomy, including lower rates of pneumonia and other pulmonary complications [16].

However, when comparing different minimally invasive techniques (MIE) (conventional MIE vs. RAMIE), the impact of technical approach appears less decisive, as demonstrated by the RAMIE trial’s findings of comparable pulmonary complication rates between these techniques [17].

Studies on Enhanced Recovery After Surgery (ERAS) protocols in esophagectomy support the advantages of restrictive fluid management, showing that reduced intraoperative fluid administration has been associated with improved postoperative outcomes [18], which is consistent with our findings that link higher intraoperative fluid balance to an increased incidence of pulmonary complications.

All other comorbidities were not associated to low or high fluid balance. But our analysis is of retorspective nature, and therefore also underpowered to detect small effect sizes. Prior to the discussion of the secondary outcomes, we clearly aim to state that the direction of the effect may warrant further investigation in larger or prospectively designed studies for POAF, anastomic leakage and AKI.

### POAF

Intraoperative fluid management appears to play a complex and nuanced role in the development of POAF in patients undergoing esophagectomy. While excessive fluid administration has been associated with an increased risk of POAF – potentially due to atrial distension, elevated cardiac preload and arrhythmogenic remodeling [19] - our findings did not find an association of POAF and fluid.

In our cohort, reduced intraoperative fluid administration may have contributed to POAF through alternative physiological mechanisms, including hypovolemia-induced sympathetic activation. Another potential explanation for the observed protective effect of intraoperative fluid balance in our study may lie in the patient-specific factors. Notably, 42.5% of patients in our cohort had pre-existing arterial hypertension, a condition commonly associated with left ventricular hypertrophy and atrial remodeling. These structural changes may predispose individuals to POAF and increase their dependence on adequate intravscular volume. Nevertheless, it should be noted that lower intraoperative fluid administration often necessitates increased use of vasopressors, particularly norepinephrine, to maintain hemodynamic stability. These agents, while effective in preserving perfusion pressure, are known to have arrhythmogenic properties and may contribute to the development of cardiac rhythm disturbances, including (POAF), particularly in the early postoperative period. In a recent study, postoperative atrial fibrillation (POAF) was identified as a major predictor of morbidity and mortality, being strongly associated with pulmonary complications, anastomotic leakage, prolonged ICU stay, and increased early mortality [20]. Therefore, reducing modifiable risk factors—such as individualised intraoperative fluid management and the optimal treatment of pre-existing cardiac comorbidities [21]—is of critical importance to prevent the occurrence of POAF.

### Anastomic leackage

A recent study from 2024 involving patients undergoing minimally invasive esophagectomy found no direct association between intraoperative fluid volume and anastomotic leakage. However, using the Cochrane-Armitage trend test, the authors attempted to demonstrate a trend toward higher leakage rates with increasing infusion volumes. The estimated cutoff for total intraoperative fluid volume predictive of anastomotic leakage was 3,792 mL, albeit with limited sensitivity (60%)[22]. In line with the hypothesis that fluid overload may impair anastomotic healing, Glatz et al. reported that postoperative fluid accumulation was associated with adverse surgical outcomes. Notably, the median intraoperative fluid rate in their cohort was 13 ml/kg/h—substantially higher than in our study population [23]. Similiarly, Kubo et al. demonstrated that a higher fluid balance on postoperative day (POD) 1 (≥ 3000 mL) was associated with an increased incidence of anastomotic leakage in patients undergoing minimally invasive esophagectomy [15]. These findings support the notion, that excessive fluid administration in the immediate perioperative period may promote tissue edema, impair healing, and increase the risk of anastomotic leakage.

In our cohort, interestingly the incidence of anastomotic complications followed an inverted U-shaped pattern: complication rates increased from the lowest to the mid-range fluid volume categories, peaking in the 4.7–8.12 mL/kg/h range, and declined again at both lower and higher fluid levels. This suggests that neither strict fluid restriction nor liberal administration alone sufficiently accounts for the risk of anastomotic leakage in this retrospective analysis. Rather, the relationship likely reflects a complex interplay between fluid balance, tissue perfusion, hemodynamic stability and the physiological stress response to major surgery. While the result regarding anastomotic complications in our cohort is not conclusive, the observed numerical trend may point toward a potential association between increased intraoperative fluid balance and impaired anastomotic healing as seen in Table 4.

### AKI

In our cohort, intraoperative fluid volume was not associated with the development of acute kidney injury (AKI), and no clear volume-dependent trend—neither increasing nor decreasing—was observed.

Previous studies have reported inconsistent results. Shin et al. described a U-shaped relationship between fluid volume and postoperative complications, including AKI, in non-cardiac surgery, indicating that both excessive and overly restrictive fluid strategies can be harmful [1]. In contrast, Abd El Aziz et al.. found no association between intraoperative fluid volume and AKI in colorectal surgery [6], indicating that factors other than intraoperative fluid balance—such as hemodynamic stability, avoidance of intraoperative hypotension, and vigilant renal monitoring—may play a more decisive role in AKI development [6]. In their cohort analysis, Salahudin et al. demonstrated that fluid overload is an independent risk factor for the development of acute kidney injury [24].

In our cohort, the observed tendency toward lower intraoperative fluid volumes in patients who developed AKI may reflect a clinically relevant signal. Too restrictive intraoperative fluid strategies have been associated with reduced renal perfusion and increased risk of ischemic kidney injury in prior studies [4].

In the context of RAMIE, these findings highlight the importance of a balanced fluid strategy to reduce the risk of complications, especially pulmonary morbidity, while acknowledging that the prevention of AKI may rely more heavily on maintaining hemodynamic stability than on absolute fluid volume. The relationship between intraoperative fluid management and AKI likely reflects a complex physiological interplay: to ensure sufficient renal perfusion is essential, yet excessive fluid administration may promote renal venous congestion and increase interstitial pressure, both of which can compromise renal function [25].

### Delirium

Regarding the secondary endpoints of our study we assessed the association between intraoperative fluid balance and the ocurrence of postoperative delirium. Postoperative delirium is a frequent and clinically significant complication following major surgery, including esophagectomy, and may be influenced by both the composition and volume of intraoperative fluids. Hydroxyethyl starch, in particular, has been identified as an independent risk factor for early postoperative delirium [22]. Beyond fluid type, fluid volume itself plays a critical role: while excessive fluid administration is associated with pulmonary complications, insufficient volume may result in tissue hypoperfusion and ischemia. In our cohort, a positive association was observed between higher intraoperative fluid volumes (ml/kg/h) and the incidence of postoperative delirium, suggesting that fluid overload may contribute to its pathogenesis.

Although our results did not reveal an association between intraoperative fluid balance and the severity of postoperative complications—as defined by major complications according to the Clavien-Dindo classification—or with ICU length of stay, previous studies have suggested more complex non-linear relationships. A retrospective multicenter study published in 2021 reported a U-shaped association between intraoperative fluid volume and the incidence of composite postoperative complications [10]. Similarly, a large hospital registry study in patients undergoing non-cardiac surgery found a U-shaped relationship between fluid volumes and renal complications, along with increased odds of respiratory complications in patients receiving more liberal fluid volumes [1].

Notably, the small subgroup with near-zero net intraoperative balance (*n* = 6) exhibited the most favorable outcome profile. While severely underpowered for inference, this observation is hypothesis-generating and aligns with reports advocating conservative or zero-balance targets in esophagectomy within ERAS pathways. Future randomized trials should explicitly test zero-balance versus individualized targets in RAMIE.

This study benefits from several methodological strengths. The inclusion of a homogeneous patient cohort with standardized intraoperative fluid management and surgical techniqueenhances the internal validity of the findings. By employing weight-adjusted fluid volumes and analyzing fluid administration as a continuous variable, the statistical models allowed for more precise estimations of associations. In particular, the detailed statistical analysis, including the presentation of predicted probabilities, offers valuable clinical insights and may serve as a practical tool to translate these findings into clinical decision-making. Furthermore, the trend analyses allowed for the identification of potential non-linear relationships, provided a more nuanced understanding of fluid dynamics in the context of RAMIE. We also aimed to present a comprehensive assessment of major postoperative complications and performed an integrative analysis of intraoperative fluid therapy in relation to these outcomes.

In this retrospective analysis, fluid administration ranged from − 2 ml/kg/h to 15 ml/kg/h, which is overall lower than the volumes used in previous studies that clearly demonstrated a negative association between high fluid balance and patient outcomes. Restricted volume therapy has entered clinical practice. Therefore, we now have to perform trials with the fluid balance target that shows the optimum for all primary, and secondary outcomes. Our results also underscore the limitation of fixed fluid thresholds and highlight the need for more individualized, risk-adapted fluid strategies. Future research should adopt a patient-centered approach carefully balancing the potential risks and benefits of liberal versus restrictive fluid administration. Incorporating patient-specific factors—such as BMI, ASA classification, and sex—into predictive models may facilitate the development of more tailored and effective fluid management protocols in esophageal surgery. A fixed fluid target does not fit all. We recommend patient-specific, complication-oriented fluid strategies in RAMIE.

Given the retrospective design, several relevant perioperative process variables were not systematically recorded: the specific monitoring and goal targets guiding fluid and vasopressor therapy, vasoconstrictor dose trajectories, phase-specific (abdominal vs. thoracic) intraoperative fluid balance, and use of epidural analgesia, which may affect vasomotor tone and fluid requirements. These factors may confound the observed associations and warrant prospective collection in future studies.

## Conclusion

This study provides compelling evidence that intraoperative fluid management significantly influences postoperative outcomes in patients undergoing robotic-assisted minimally invasive esophagectomy (RAMIE). Our findings show a clear dose-dependent association between higher intraoperative fluid volumes and an increased incidence of pulmonary complications, with the risk nearly fivefold higher at the upper fluid range.

Intraoperative fluid volume during RAMIE showed outcome-specific associations: higher volumes were linked to increased pulmonary morbidity, and anastomotic complications followed a non-linear pattern with a peak at moderate volumes.

## Data Availability

The datasets used and analyzed during the current study are available from the corresponding author upon reasonable request.
